# Disparities in Risk Factors Associated with Obesity between Zanzibar and Tanzania Mainland among Women of Reproductive Age Based on the 2010 TDHS

**DOI:** 10.1155/2016/1420673

**Published:** 2016-09-18

**Authors:** Edwin Paul, Abdalla H. Mtumwa, Julius Edward Ntwenya, Said A. H. Vuai

**Affiliations:** ^1^Department of Statistics, The University of Dodoma, P.O. Box 338, Dodoma, Tanzania; ^2^Department of Public Health, The University of Dodoma, P.O. Box 395, Dodoma, Tanzania; ^3^Department of Chemistry, University of Dodoma, P.O. Box 338, Dodoma, Tanzania

## Abstract

The occurrence of overweight and obesity has serious health implications. The 2010 Tanzania Demographic and Health Survey data set was reanalysed to compare the prevalences of overweight and obesity between Mainland Tanzania and Zanzibar and to determine how demographic factors can predict overweight and obesity across the United Republic of Tanzania. About 7.92% of the Tanzanian women of reproductive age were obese, 15% were overweight, and 11.5% were underweight. Women from Mainland Tanzania (6.56%) were significantly less likely (AOR = 0.66, 95% CI: 0.53–0.82) to be affected by obesity as compared to women from Zanzibar (12.19%). The common predictors of obesity in Mainland Tanzania and Zanzibar were wealth index, marital status, and age. Whereas the place of residence and education level emerged as predictors of obesity in the Mainland Tanzania alone, the number of meals per day did so in Zanzibar. Most importantly, Zanzibar had a greater prevalence of obesity compared to Mainland Tanzania.

## 1. Introduction

Overweight and obesity are the result of excessive fat accumulation which leads to impaired health. Nearly two billion adults worldwide are overweight, and of these, more than half a billion are obese [1]. The global prevalence of overweight in the year 2014 was around 39% among adults aged 18 years or older, being 39% for men and 40% for women. About 13% were obese (BMI ≥ 30 kg/m^2^), of which 11% were men and 15% were women [1, 2].

The global prevalence of obesity has been increasing. The number of overweight individuals was estimated to increase from 937 million in 2005 to 2.16 billion in 2030 and the number of obese individuals from 396 million to 1.12 billion [3]. Obesity contributes significantly to the global burden of noncommunicable disease and is responsible for 44% of diabetes, 23% of the ischemic heart disease, and between 7% and 41% of certain cancer burdens [1]. The coexistence of overweight and obesity as well as undernutrition in developing countries, also termed the morbidity transition, places African countries under more burden to address nutrition and other health-related challenges. The current demographic transition and food consumption shift associated with behavioral changes are main drivers for the existence of the double burden malnutrition. In recent years, overweight and obesity have become one of the five leading threats of global mortality [4]. It is approximated that at least 2.8 million adults die every year due to either overweight or obesity [2].

Factors such as diet, level of physical activity, and age have widely been reported to influence obesity [5, 6]. Obesity increases with age which may be explained by decreases in physical activity and metabolic activities in older adults [7]. Other risk factors positively associated with obesity include marital status, high educational level, alcohol use, and high socioeconomic status [8].

Few surveys have been conducted in Tanzania to determine the prevalence and risk factors associated with overweight and obesity among the adult population [6]. The Tanzania Demographic and Health Survey (TDHS) is the main source of obesity and overweight data in Tanzania [9]. There is an increasing level of obesity and overweight in Tanzania at around 2.7% and 5.3%, respectively, between 1991 and 1992 when the first survey was implemented [9]. A few studies that have been implemented in Tanzania were district-specific and focused on a small population making it difficult to generalize about issues related to prevalence and risk factors. A study by Kinabo et al. [10] conducted in rural areas of Morogoro and Iringa regions showed that the prevalence of overweight and obesity was 9% in males and 23.3% among females. In other selected areas of Tanzania, the prevalence of overweight and obesity was reported as 16% and 6%, respectively [11]. Further evidence shows that obesity was more common in women and has become a major health concern [12].

More studies are needed to understand the nature and severity of obesity in developing countries. In addition, the evidence generated will help to develop interventions that are culturally sensitive. Therefore, the reanalysis of the national data sets will be a good starting point especially in situations where the design and implementation of larger national representative surveys would be difficult. The present analysis sought to illuminate the existing risk factors and disparities in obesity prevalence between Mainland Tanzania and Zanzibar.

## 2. Methods

### 2.1. Source of Data

Data from the 2010 TDHS data set was utilized to perform secondary data analysis on the prevalence of obesity and risk factors among nonpregnant women of reproductive age. The methods are those used in the TDHS and are described briefly below. A total of 6,642 households were studied and these gave rise to a sample size of 9,131 women: 6,933 women from Mainland Tanzania and 2,198 from Zanzibar. The household member's data file was used to capture all data needed including weight, height, and age. Data collection was done using the Household Questionnaire (woman's weight and height) and the Women's Questionnaire (meals per day, wealth index, education level, marital status, age, use of cooking oil, and place of residence). Weight and height were measured using standard anthropometric procedures. BMI was calculated from measured weight and height as a ratio of weight (Kg) and height (M^2^). Selected predictors for obesity include wealth index, age, and consumption of cooking oil. The wealth index and age were selected as predictors because there is association between those predictors with obesity [9]. It is also known that there is association between eating patterns such as number of meals eaten per day and consumption of certain foods with obesity [13]. Use of cooking oil gives an indication of the extra energy taken from fat, hence a possibility for higher obesity risk among users in the presence of other predisposing factors. It was also important to illuminate association between obesity and other predictors, namely, education level, marital status, and place of residence. The inclusion of these demographic variables was relevant at capturing how different groups within our societies are affected by overweight and obesity to inform policy decision-makers.

### 2.2. Sample Size and Sampling Procedure

A representative probability sample of 10,300 households was selected for the 2010 TDHS. The sample was selected in two stages. In the first stage, 475 clusters were selected from a list of enumeration areas according to the 2002 Population and Housing Census. Twenty-five sample points were selected in Dar es Salaam, and 18 were selected in each of the other twenty regions in Mainland Tanzania. In Zanzibar, 18 clusters were selected in each region for a total of 90 sample points. In the second stage, a complete household listing was carried out in all selected clusters between July and August, 2009. The households were then systematically selected for participation in the survey. Twenty-two households were selected from each of the clusters in all regions, except Dar es Salaam where 16 households were selected in each sample point. All women aged 15–49 years and who were either the permanent residents or visitors present in the households on the night before the survey were eligible to be included in the 2010 TDHS sample. In the interviewed households, 10,522 eligible women from 7,428 households were identified for individual interview. In our analysis, we excluded 449 subjects whose BMIs were not recoded and 942 pregnant women which resulted in decrease in households from 7,428 to 6,642.

### 2.3. Data Analysis

The obesity variable was derived by computing the BMI. The BMI (kg/m^2^) was used to classify participants as underweight, normal, overweight, and obese [14]. The generated BMI categories were then recorded to generate a binary variable with two levels (i.e., obese and not obese). Women having a BMI of 30 kg/m^2^ and above were considered obese, while women with a BMI of less than 30 kg/m^2^ were classified as not obese. The generated binary obesity variable was used throughout for descriptive, bivariable, and multivariable analyses. Categorical variables are presented as the number of observations and its corresponding percentage. The chi square (*χ* ^2^) test was used to find associations between categorical variables. In many regression applications, observations have some kind of clustering, with observations within cluster and 95% tending to be correlated. In our context, the unit of sampling was the household; at last, all the eligible women in the sampled households were sampled thus bringing in a cluster (household) of household members who are likely to have similar background characteristics. Thus, the assumption of independence of observations within a cluster does not hold because the subjects share the same cluster. Therefore, for a clustered binary outcome, a Generalized Estimating Equation (GEE) becomes a candidate model to account for correlation among the subjects with a cluster [15]. In this case, a crude odds ratio (OR) at 95% confidence was calculated using univariable Generalized Estimating Equation to estimate the association between obesity and the independent variables. Multivariable modeling was employed to determine which factors were associated with obesity, while the association was adjusted for other variables. *p* values were estimated by two-sided tests. Statistical significance was set at a *p* value of less than 0.05.

### 2.4. Ethical Issues

Tanzania's National Institute for Medical Research (NIMR), the Zanzibar Medical Ethics and Research Committee (ZAMREC), the Institutional Review Board of ICF International, and the Centers for Disease Control and Prevention in Atlanta gave ethical clearance to the study. The secondary analysis of the 2010 TDHS data got approval of the National Bureau of Statistics.

## 3. Results

### 3.1. Characteristics of the Respondents

Demographic characteristics of the 9,131 women from 6,642 surveyed household are presented in Table 1. There were 6,933 women from Mainland Tanzania and 2,198 from Zanzibar. About 74% of respondents lived in rural areas. Tanzania Mainland had a significantly higher proportion of rural respondents (74.89%, *p* < 0.0001) compared to Zanzibar (69.84%). Zanzibar had the highest proportion of women grouped in the richest wealth category (39.5%, *p* < 0.0001) compared to Mainland Tanzania. Most women had a primary education (57.5%) and fewer had no formal education (18%). The proportion of women with no primary education was found to be significantly (*p* < 0.0001) higher in Mainland Tanzania (18.7%) compared with Zanzibar (15.9%). About 60% of women were either married or cohabitating, with the proportion of married or cohabitating women being significantly higher in Mainland Tanzania (62%, *p* < 0.0001) than in Zanzibar (51.9%). The majority (32.7%) of the surveyed women belonged to the 20–29 years' age category. About 79.3% of Tanzanian women reported using oil during cooking. The largest proportion (84.4%, *p* < 0.0001) of the women in Mainland Tanzania added oil to their meals compared to Zanzibar (63.3%). More than 60% of the women in Mainland Tanzania and Zanzibar consumed three or four times meals per day, and 2% eat one meal per day in both Mainland Tanzania and Zanzibar.

### 3.2. Prevalence of Obesity among Nonpregnant Women

**Figure 1:**
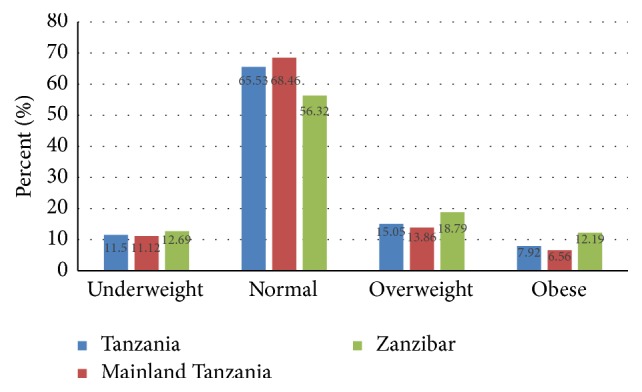
Overweight and obesity prevalence among nonpregnant women in Tanzania.

About 15% of the surveyed women were overweight and 7.92% were obese (Figure 1). The majority (65.53%) had their BMI within the normal range and 11.5% were underweight. In Mainland Tanzania, 13.86% were overweight and 6.56% were obese. In Zanzibar, the proportion of overweight women (18.79%) and obese (12.19%) was found to be higher compared to that of women in Mainland Tanzania.

### 3.3. Distribution of Obesity Prevalence among Nonpregnant Women by Baseline Characteristics and State

The results of the bivariable analysis using Pearson's chi square test showed significant associations between all explanatory variables and women's obesity in Mainland Tanzania (Table 2). In Zanzibar, all the underlying characteristics except women's education (*p* = 0.229) were significantly associated with women's obesity. Obesity prevalence among women living in an urban setting was significantly higher compared to women in rural areas in both Mainland Tanzania and Zanzibar. The prevalence of obesity among women in urban settings was higher in Zanzibar (17%) compared to Mainland Tanzania (14.4%). Similarly, most women belonging to the richest wealth categories were obese regardless of the state. Other significant determinants of obesity included education, marital status, age, use of cooking oil, and number of meals per day.

### 3.4. Prevalence of Obesity among Nonpregnant Women by Age Groups

**Figure 2:**
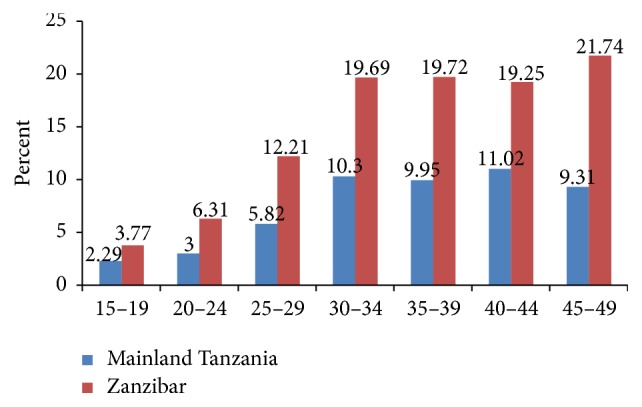
Percentage of women with obesity by age groups in Tanzania: Mainland and Zanzibar.

The prevalence of obesity was shown to increase with age to around 30 to 34 years and appeared to be almost constant from 30 to 44 years of age. In each age category, the prevalence of obesity was higher among women in Zanzibar compared to women in Mainland Tanzania (Figure 2).

### 3.5. Distribution of Obesity Prevalence by Regions in Tanzania

**Figure 3:**
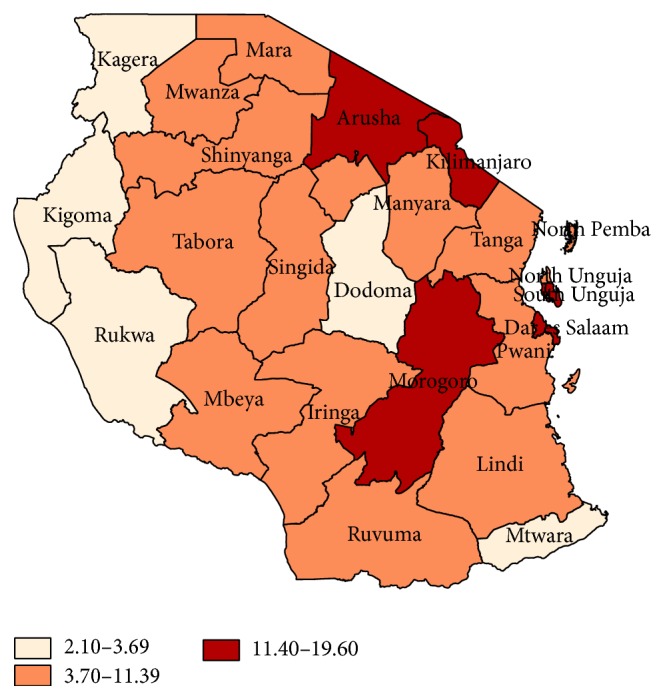
Obesity prevalence categories by region of residence among nonpregnant women of reproductive age.

A map depicts that western regions of Tanzania had the lowest prevalence of obesity among nonpregnant women of reproductive age ranging from 2.1 to 3.69% (Figure 3). Other regions with the lowest obesity prevalence included Dodoma and Mtwara both in the Mainland Tanzania. The prevalence of obesity ranged from 3.7% to 11.39% for most regions. Major regions and cities (namely, Dar es Salaam, Arusha, Morogoro, Kilimanjaro, and Town west-Unguja) had the highest prevalence of obesity ranging from 11.4% to 19.6%. Obesity prevalence in Zanzibar among nonpregnant women ranged from 3.7% to 19.6%. Most women in the Unguja Island were obese with prevalence between 11.4% and 19.6%.

### 3.6. Odds of Obesity among Nonpregnant Women in Tanzania

As indicated in Methods, GEE with exchangeable working correlation structure was applied to find the important risk factors associated with obesity among nonpregnant women of reproductive age. The results of the fitted models for Mainland Tanzania, Zanzibar, and pooled sample are presented in Tables 3 [Table tab4]–5.

### 3.7. Factors Associated with Obesity among Women of Reproductive Age in Mainland Tanzania

In Mainland Tanzanian women, the results of univariable analysis showed that all independent variables were significantly associated with obesity (Table 3). Nevertheless, in multivariable analysis, the use of oil for cooking food and number of meals per day were no longer significantly related to obesity. Place of residence, wealth index, education level, marital status, and age of the respondents were important independent risk factors of obesity among women of reproductive age. The results of multivariable analysis presented in Table 3 revealed that urban women (AOR = 1.37, 95% CI: 1.03–1.81) were significantly more likely to be obese compared to their rural counterparts. The odds of obesity among women in Mainland Tanzania were positively related to family wealth index. Subjects in the middle (AOR = 1.84, 95% CI: 1.12–3.03), richer (AOR = 3.23, 95% CI: 1.99–5.24), and richest (AOR = 8.93, 95% CI: 5.32–15.00) wealth index categories were at significantly greater odds of being obese in comparison to subjects in the poorest category. Regarding education level, women with secondary or higher education had a significantly increased odds of obesity compared to women with no formal education (AOR = 1.57, 95% CI: 1.02–2.44). The odds of obesity among women with a primary education were not significantly different from the odds of women with no formal education (AOR = 1.36, 95% CI: 0.95–1.95). Married and cohabitating women (AOR = 2.44, 95% CI: 1.63–3.66) had significantly greater probability of obesity compared to never married women. Divorced and widowed women had a significantly greater odds of obesity (AOR = 1.74, 95% CI: 1.06–2.85) compared to never married women. Older women (30–39; 40–49) had significantly greater odds of being obese compared to younger adults (15–19) [30–39 (AOR = 3.53, 95% CI: 2.16–5.76); 40–49 (AOR = 4.18, 95% CI: 2.52–6.91)].

### 3.8. Factors Associated with Obesity among Nonpregnant Women of Reproductive Age in Zanzibar

In contrast to subjects in Mainland Tanzania where education level was significantly associated with obesity, in Zanzibar, the results of univariable analysis (Table 4) revealed that education level was not significantly associated with obesity among women of reproductive age. Other independent variables, namely, place of residence, wealth index, marital status, age, the use of cooking oil, and meals per day, were found to be significant. The results of multivariable analysis (Table 4) showed that wealth index, marital status, age, and number of meals eaten per day were significantly associated with obesity among women in Zanzibar. Women belonging to the richest wealth index category (AOR = 2.36, 95% CI: 1.01–5.52) were significantly more likely to be obese compared to women from the poorest category. Divorced or widowed (AOR = 2.44, 95% CI: 1.37–4.32) and married or cohabited women (AOR = 1.12, 95% CI: 1.27–3.54) were at significantly greater odds of being obese compared to women who never married. Women aged 30–39 (AOR = 3.10, 95% CI: 1.62–5.92) and 40–49 (AOR = 3.52, 95% CI: 1.81–6.82) were significantly at greater odds of being obese than young women. Another predictor associated with obesity among women in Zanzibar was the number of meals consumed per day. Women who consumed meals two times per day (AOR = 0.67, 95% CI: 0.48–0.92) had significantly lower odds of obesity than women consuming three to four meals.

### 3.9. Factors Associated with Obesity among Nonpregnant Women in Tanzania (Pooled Sample)

Women from Mainland Tanzania were significantly less likely (AOR = 0.66, 95% CI: 0.53–0.82) to be obese compared to women from Zanzibar (Table 5). Likewise, the results of GEE for pooled sample showed that the likelihood of a woman being obese increased with an increase in the wealth index. Women in the middle (AOR = 1.79, 95% CI: 1.17–2.75), richer (AOR = 2.99, 95% CI: 1.99–4.50), and richest (AOR = 7.24, 95% CI: 4.66–11.24) wealth categories were at higher risk of being obese than women in the poorest category. The risk of obesity among women in poorer wealth index (AOR = 1.33, 95% CI: 0.85–2.07) was not significantly different from that of the poorest women. Widowed or divorced women and married or cohabited women were more likely (AOR = 1.95, 95% CI: 1.34–2.84, and AOR = 2.27, 95% CI: 1.66–3.11) to be affected by obesity than never married women. Another important predictor was age, with women aged 30–39 (AOR = 3.29, 95% CI: 2.23–4.84) and 40–49 (AOR = 3.76, 95% CI: 2.53–5.59) having higher odds of being obese than women aged 15–19. However, the odds of being obese for women aged 20–29 were not significantly different from those of women of age between 15 and 19 years (AOR = 1.37, 95% CI: 0.95–1.96). Women who consumed two meals per day were significantly less likely (AOR = 0.73, 95% CI: 0.59–0.91) to be obese compared to those who consumed three to four meals per day.

## 4. Discussion

Our analysis has shown that the prevalence of obesity was higher among the studied population of women of reproductive age with 7.92% being obese. The observed prevalence in this case was 5.08% lower than the overall global prevalence (13%) of obesity [1]. However, the rate of obesity is increasing. The present analysis is meant to illuminate the existence of obesity among women and to provoke policy actions in order to improve the health of women. For many years, developing countries have been experiencing undernutrition but, recently, overweight and obesity rates are growing. More women are now at risk of noncommunicable diseases and associated comorbidities including hypertension, diabetes, cancer, stroke, and ischemic heart disease [16]. Our observation concurs with findings in Spain that reported an increase in the prevalence of overweight and obesity prevalence from 5.1 to 8.3% in adults for the period prior to 2010 [17]. The results compare well with the observations made among women adults in Colombia, which reported an increase in obesity prevalence (17% in 2005 and 20% in 2010) and its burden is shifting towards the poor and urban populations [18]. This increase was found in sub-Saharan Africa as well as in a study conducted in Kenya that found a 5% annual increase in obesity [19].

There was variation in obesity prevalence between Mainland Tanzania and Zanzibar, with Zanzibar having almost six percent higher prevalence compared to Mainland Tanzania. The existence of the higher level of obesity in Zanzibar, which was almost twice the level in the Mainland Tanzania, shows the severity of obesity and its contribution to noncommunicable diseases landscape in Tanzania. This may be explained by Zanzibar becoming more developed and undergoing the nutrition transition: a greater proportion of inhabitants of Zanzibar consume three or more meals per day, consume meat and fish, live within 2 km of a health facility, are in the highest wealth quintile, and are more educated [20]. Additionally, a smaller proportion of people in Zanzibar suffer from HIV (Zanzibar = 1.1%, Mainland Tanzania = 6.3%). In addition, the determinants of obesity such as lifestyle behaviors may have had varying contribution to obesity prevalence among the two parts of the United Republic of Tanzania. There is a need for further studies and there would be a need to employ mixed methods to explore social and environmental determinants of obesity in Tanzania. Thus, the nutrition transition, the demographic transition, and the epidemiological transition might be more prominent in Zanzibar compared to Mainland Tanzania. Regions identified with more obesity prevalence also experience more human development expressed in terms of per capital gross net product, quality of life, and poverty reduction [20]. For example, the 2014 human development report classified Kilimanjaro, Dar es Salaam, and Arusha as medium income regions [21].

Our analysis has attempted to determine the potential factors contributing to obesity among women of reproductive age. As expected, urban women had greater odds of being obese. A higher prevalence of obesity among urban women was reported in Benin West Africa as well [22]. However, the level of obesity in rural areas was equally high. This observation suggests that obesity has conquered the rural barrier and is likely to persist if no well-prepared measures to address it in holistic terms are put in place. Studies done in rural areas of Tanzania that focused on small populations such as districts have reported higher levels of obesity and overweight. In a study done among women in a rural setting, the prevalence of overweight was 16% and that of obesity was 6% [11]. A similar study done in a rural area reported a 14.2% overweight prevalence and 3.2% obesity level [10]. Studies on obesity occurrence in Tanzania in urban centers have shown that obese individuals rarely consider obesity to be a problem. Among overweight men, about 22% perceived themselves as overweight or obese compared to 38% of overweight women who perceived themselves as overweight or obese [23]. An increasing level of obesity among rural women can be explained by increased access to processed foods, changes in cooking styles, and declining level of physical activity as a result of declining participation in farming activities and the general demographic transition. Similarly, higher prevalence of obesity in urban areas could be explained by an increase in sedentary lifestyle associated with urbanization such as physical activity level, income, and meal sizes [24].

Women belonging to households that were categorized wealthier had extra odds of being obese. The existence of higher level of obesity among wealthier families indicates that the wealthier did not opt for healthier eating patterns and were probably not adhering to healthier lifestyle behaviors. For the wealthier in many parts of Tanzania, walking, for example, is not adequately conceived an opportunity to do physical exercise; rather, it is considered as lack or inability to afford a more decent means of transport. In a study conducted in Turkey, Ergin et al. [25] showed a 46% overweight prevalence among women and a significant increase in obesity among the highest wealth groups. Greater odds of being obese were observed among married and cohabiting women, older women, women who consumed three to four meals a day, and women who used added oil in form of fat. The possible explanation for the married women could be the presence of a male and the perceived peace of mind. The other explanation could be an additional income among married individuals. The prevalence of obesity was higher among older women. The increase in obesity prevalence with age has widely been reported [20, 26]. The odds of being obese were five times greater in subjects aged 55 years or older compared to the youngest subjects (OR (95% CI) = 5.1 (2.5–10.4), *p* < 0.001) and were reported in a study done among adults in Kinondoni District of Tanzania [6]. Similarly, among Jordanian women of reproductive age based on three DHSs, 2002–2012 results show that the prevalence of obesity increased by ten percent for every additional year of age [24]. The reason for increasing obesity with age is related to sedentary lifestyle and reduced metabolic rates [27, 28].

Our analysis has also showed that intake of three or more meals was related to obesity. The dietary guideline recommends that adults take at least three meals a day. The current observation may contradict the ongoing efforts to encourage people to eat, especially among the undernourished communities; therefore, they may need to be interpreted with caution. Though it is recommended to eat at least three meals per day, people should choose a more balanced diet. It is important, therefore, to encourage households to consume diversified diets that are nutrient rich.

It should be noted that in this study the response variable BMI (kg/m^2^) was measured during interview. Though the predictors (meals per day, wealth index, education level, marital status, age, and use of cooking oil) were self-reported during interview [9], the limitations of self-report in the survey include overestimating and incorrect self-reporting which may be caused by recall, social desirability, and self-observation bias [29, 30].

## 5. Conclusion

The prevalence (7.92%) of obesity was higher among women of reproductive age. Zanzibar had higher prevalence (12.19%) of obesity compared to Mainland Tanzania (6.56%). The prevalence of obesity appeared to be greater among Zanzibar women in all the age groups in our study. The common predictors of obesity in Mainland Tanzania and Zanzibar were wealth index, marital status, and age. Whereas the place of residence and education level emerged as predictors of obesity in the Mainland Tanzania alone, the number of meals per day did so in Zanzibar. Most importantly, Zanzibar had higher prevalence of obesity compared to Mainland Tanzania.

## Figures and Tables

**Table 1:** Baseline characteristics of the 9131 nonpregnant women surveyed in Mainland Tanzania and Zanzibar.

Variable	Total sample	Mainland Tanzania	Zanzibar	*p* value
	*n* (%)	*n* (%)	*n* (%)	
*Place of residence*				
Urban	2,404 (26.33)	1,741 (25.11)	663 (30.16)	
Rural	6,727 (73.70)	5,192 (74.89)	1,535 (69.84)	<0.0001
*Wealth index*				
Poorest	1,449 (15.90)	1,324 (19.10)	125 (5.70)	
Poorer	1,642 (18.00)	1,406 (20.30)	236 (10.70)	
Middle	1,680 (18.40)	1,384 (20.00)	296 (13.50)	
Richer	2,078 (22.80)	1,406 (20.3)	672 (30.60)	
Richest	2,282 (25.00)	1,413 (20.4)	869 (39.50)	<0.0001
*Education level*				
No education	1,646 (18.00)	1,296 (18.7)	350 (15.90)	
Primary	5,252 (57.50)	4,636 (66.90)	616 (28.00)	
Secondary and higher	2,226 (24.40)	995 (14.40)	1,231 (56.00)	<0.0001
*Marital status*				
Never married	2,661 (29.10)	1,808 (26.10)	853 (38.80)	
Divorced/widowed	983 (10.80)	783 (11.30)	200 (9.10)	
Married/cohabiting	5,440 (59.60)	4,299 (62.00)	1,141 (51.90)	<0.0001
*Age (years)*				
15–19	2,069 (22.70)	1,485 (21.40)	584 (26.60)	
20–29	2,983 (32.70)	2,284 (32.90)	699 (31.80)	
30–39	2,368 (25.90)	1,896 (27.30)	472 (21.50)	
40–49	1,711 (18.70)	1,268 (18.30)	443 (20.20)	<0.0001
*The use of cooking oil*				
No	1,891 (20.70)	1,084 (15.60)	807 (36.70)	
Yes	7,240 (79.30)	5,849 (84.40)	1,391 (63.30)	<0.0001
*Meals per day*				
1 time	183 (2.00)	141 (2.00)	42 (1.90)	
2 times	3,404 (37.30)	2,565 (37.00)	839 (38.20)	
3 to 4 times	5,540 (60.70)	4,223 (60.90)	1,317 (59.90)	0.602

**Table 2:** Distribution of obesity prevalence among nonpregnant women by baseline characteristics and state.

Variable	Mainland Tanzania			Zanzibar		
	Total	Number of obese women (%)	*p* value	Total	Number of obese women (%)	*p* value
*Place of residence*						
Urban	1741	251 (14.40)		663	113 (17.00)	
Rural	5192	204 (3.90)	<0.0001	1535	155 (10.10)	<0.0001
*Wealth index*						
Poorest	1324	25 (1.90)		125	9 (7.20)	
Poorer	1406	35 (2.50)		236	17 (7.20)	
Middle	1384	51 (3.70)		296	22 (7.40)	
Richer	1406	93 (6.60)		672	63 (9.40)	
Richest	1413	251 (17.80)	<0.0001	869	157 (18.10)	<0.0001
*Education level*						
No education	1296	40 (3.10)		350	37 (10.60)	
Primary	4636	323 (7.00)		616	86 (14.00)	
Secondary and higher	995	92 (9.20)	<0.0001	1231	144 (11.70)	0.229
*Marital status*						
Never married	1808	52 (2.90)		853	41 (4.80)	
Divorced/widowed	783	52 (6.60)		200	37 (18.50)	
Married/cohabiting	4299	348 (8.10)	<0.0001	1141	190 (16.70)	<0.0001
*Age (years)*						
15–19	1485	34 (2.30)		584	22 (3.80)	
20–29	2284	99 (4.30)		699	62 (8.90)	
30–39	1896	192 (10.10)		472	93 (1970)	
40–49	1268	130 (10.30)		443	91 (20.50)	<0.0001
*The use of cooking oil*						
No	1084	24 (2.20)		807	76 (9.40)	
Yes	5849	431 (7.40)	<0.0001	1391	192 (13.80)	0.002
*Meals per day*						
1 time	141	7 (5.00)		42	1 (2.40)	
2 times	2565	85 (3.30)		839	69 (8.20)	
3 to 4 times	4223	363 (8.60)	<0.0001	1317	198 (15.00)	<0.0001

**Table 3:** Crude and adjusted odds ratios of obesity among nonpregnant women in Mainland Tanzania.

Variable	Univariate analysis			Multivariate analysis		
	OR (se)	95% CI	*p* value	AOR (se)	95% CI	*p* value
*Place of residence*						
Urban	4.13 (0.41)	[3.40, 5.01]	<0.0001	1.37 (0.20)	[1.03, 1.81]	0.0310
Rural	Reference			Reference		
*Wealth index*						
Poorest	Reference			Reference		
Poorer	1.33 (0.35)	[0.79, 2.22]	0.2826	1.27 (0.34)	[0.75, 2.14]	0.3769
Middle	1.99 (0.49)	[1.23, 3.22]	0.0052	1.84 (0.47)	[1.12, 3.03]	0.0165
Richer	3.69 (0.85)	[2.35, 5.79]	<0.0001	3.23 (0.80)	[1.99, 5.24]	<0.0001
Richest	11.24 (2.39)	[7.41, 17.04]	<0.0001	8.93 (2.36)	[5.32, 15.00]	<0.0001
*Education level*						
No education	Reference			Reference		
Primary	2.34 (0.40)	[1.68, 3.26]	<0.0001	1.36 (0.25)	[0.95, 1.95]	0.0882
Secondary and higher	3.13 (0.61)	[2.13, 4.60]	<0.0001	1.57 (0.35)	[1.02, 2.44]	0.0415
*Marital status*						
Never married	Reference			Reference		
Divorced/widowed	2.57 (0.52)	[1.72, 3.82]	<0.0001	1.74 (0.44)	[1.06, 2.85]	0.0291
Married/cohabited	3.23 (0.51)	[2.37, 4.40]	<0.0001	2.44 (0.50)	[1.63, 3.66]	<0.0001
*Age (years)*						
15–19	Reference			Reference		
20–29	1.94 (0.41)	[1.29, 2.92]	0.0015	1.32 (0.32)	[0.83, 2.11]	0.2454
30–39	1.60 (0.19)	[1.22, 1.98]	<0.0001	3.53 (0.88)	[2.16, 5.76]	<0.0001
40–49	5.03 (0.99)	[3.42, 7.41]	<0.0001	4.18 (1.07)	[2.52, 6.91]	<0.0001
*The use of cooking oil*						
No	Reference			Reference		
Yes	3.48 (0.77)	[2.26, 5.36]	<0.0001	1.41 (0.35)	[0.87, 2.31]	0.1648
*Meals per day*						
1 time	0.55 (0.21)	[0.26, 1.16]	0.1156	1.26 (0.57)	[0.52, 3.07]	0.6126
2 times	0.36 (0.05)	[0.29, 0.46]	<0.0001	0.78 (0.11)	[0.59, 1.03]	0.0822
3 to 4 times	Reference			Reference		

Note: se stands for standard error; AOR represents adjusted odds ratios.

**Table 4 tab4:** Crude and adjusted odds ratios of obesity among nonpregnant women in Zanzibar.

Variable	Univariate analysis			Multivariate analysis		
	OR (se)	95% CI	*p* value	AOR (se)	95% CI	*p* value
*Place of residence*						
Urban	1.83 (0.25)	[1.41, 2.39]	<0.0001	1.01 (0.17)	[0.72, 1.41]	0.9586
Rural	Reference			Reference		
*Wealth index*						
Poorest	Reference			Reference		
Poorer	0.99 (0.45)	[0.41, 2.41]	0.9965	0.97 (0.45)	[0.40, 2.39]	0.9498
Middle	1.04 (0.45)	[0.44, 2.44]	0.9342	0.96 (0.43)	[0.40, 2.31]	0.9262
Richer	1.34 (0.54)	[0.61, 2.93]	0.4698	1.17 (0.48)	[0.52, 2.63]	0.7014
Richest	2.85 (1.11)	[1.33, 6.11]	0.0072	2.36 (1.02)	[1.01, 5.52]	0.0467
*Education level*						
No education	Reference			Reference		
Primary	1.36 (0.29)	[0.90, 2.05]	0.1384	1.33 (0.31)	[0.84, 2.09]	0.2227
Secondary and higher	1.11 (0.22)	[0.76, 1.62]	0.5977	1.19 (0.27)	[0.76, 1.86]	0.4555
*Marital status*						
Never married	Reference			Reference		
Divorced/widowed	4.53 (1.08)	[2.84, 7.22]	<0.0001	2.44 (0.71)	[1.37, 4.32]	0.0023
Married/cohabited	4.06 (0.75)	[2.83, 5.82]	<0.0001	2.12 (0.55)	[1.27, 3.54]	0.0039
*Age (years)*						
15–19	Reference			Reference		
20–29	2.45 (0.62)	[1.49, 4.02]	0.0004	1.51 (0.45)	[0.85, 2.70]	0.1618
30–39	6.23 (1.55)	[3.83, 10.14]	<0.0001	3.10 (1.02)	[1.62, 5.92]	0.0006
40–49	6.61 (1.58)	[4.14, 10.57]	<0.0001	3.52 (1.19)	[1.81, 6.82]	0.0002
*The use of cooking oil*						
No	Reference			Reference		
Yes	1.54 (0.23)	[1.15, 2.07]	<0.0001	1.07 (0.19)	[0.76, 1.50]	0.7070
*Meals per day*						
1 time	0.12 (0.12)	[0.02, 0.78]	0.0259	0.20 (0.18)	[0.03, 1.20]	0.0783
2 times	0.50 (0.08)	[0.37, 0.68]	<0.0001	0.67 (0.11)	[0.48, 0.92]	0.0151
3 to 4 times	Reference			Reference		

**Table 5:** Crude and adjusted odds ratios of obesity among nonpregnant Tanzanian women.

Variable	Univariate analysis			Multivariate analysis		
	OR (se)	95% CI	*p* value	AOR (se)	95% CI	*p* value
*State*						
Mainland Tanzania	0.50 (0.04)	[0.43, 0.59]	<0.0001	0.66 (0.07)	[0.53, 0.82]	0.0001
Zanzibar	Reference					
*Place of residence*						
Urban	3.18 (0.25)	[2.72, 3.71]	<0.0001	1.27 (0.14)	[1.03, 1.57]	0.0281
Rural	Reference			Reference		
*Wealth index*						
Poorest	Reference			Reference		
Poorer	1.36 (0.31)	[0.87, 2.12]	0.1719	1.33 (0.30)	[0.85, 2.07]	0.213
Middle	1.89 (0.40)	[1.25, 2.88]	0.0028	1.79 (0.39)	[1.17, 2.75]	0.0077
Richer	3.39 (0.67)	[2.30, 4.99]	<0.0001	2.99 (0.62)	[1.99, 4.50]	<0.0001
Richest	9.09 (1.69)	[6.32, 13.09]	<0.0001	7.24 (1.63)	[4.66, 11.24]	<0.0001
*Education level*						
No education	Reference			Reference		
Primary	1.71 (0.22)	[1.33, 2.19]	<0.0001	1.28 (0.18)	[0.97, 1.68]	0.0762
Secondary and higher	2.36 (0.32)	[1.81, 3.08]	<0.0001	1.28 (0.21)	[0.94, 0.12]	0.1226
*Marital status*						
Never married	Reference			Reference		
Divorced/widowed	2.94 (0.45)	[2.17, 3.98]	<0.0001	1.95 (0.37)	[1.34, 2.84]	0.0005
Married/cohabited	3.31 (0.40)	[2.60, 4.20]	<0.0001	2.27 (0.36)	[1.66, 3.11]	<0.0001
*Age (years)*						
15–19	Reference			Reference		
20–29	2.05 (0.33)	[1.49, 2.82]	<0.0001	1.37 (0.25)	[0.95, 1.96]	0.0915
30–39	5.05 (0.77)	[3.74, 6.81]	<0.0001	3.29 (0.65)	[2.23, 4.84]	<0.0001
40–49	5.48 (0.84)	[4.06, 7.40]	<0.0001	3.76 (0.76)	[2.53, 5.59]	<0.0001
*The use of cooking oil*						
No	Reference			Reference		
Yes	1.68 (0.20)	[1.34, 2.12]	<0.0001	1.09 (0.15)	[0.84, 1.42]	0.5186
*Meals per day*						
1 time	0.40 (0.14)	[0.19, 0.79]	0.0083	0.78 (0.30)	[0.37, 1.67]	0.5279
2 times	0.42 (0.04)	[0.35, 0.50]	<0.0001	0.73 (0.08)	[0.59, 0.91]	0.0042
3 to 4 times	Reference			Reference		
